# Symptom Signatures and Diagnostic Timeliness in Cancer Patients: A Review of Current Evidence

**DOI:** 10.1016/j.neo.2017.11.005

**Published:** 2017-12-16

**Authors:** Minjoung M. Koo, William Hamilton, Fiona M. Walter, Greg P. Rubin, Georgios Lyratzopoulos

**Affiliations:** ⁎ University College London, 1-19 Torrington Place, London WC1E 6BT, UK; † University of Exeter Medical School, St Luke's Campus, Heavitree Road, Exeter, EX1 2LU, UK; ‡ University of Cambridge, Primary Care Unit, Strangeways Research Laboratory, Cambridge, CB2 0SR, UK; § Institute of Health and Society, Newcastle University, Sir James Spence Institute, Royal Victoria Infirmary, Newcastle upon Tyne, NE1 4LP, UK

## Abstract

Early diagnosis is an important aspect of contemporary cancer prevention and control strategies, as the majority of patients are diagnosed following symptomatic presentation. The nature of presenting symptoms can critically influence the length of the diagnostic intervals from symptom onset to presentation (the patient interval), and from first presentation to specialist referral (the primary care interval). Understanding which symptoms are associated with longer diagnostic intervals to help the targeting of early diagnosis initiatives is an area of emerging research. In this Review, we consider the methodological challenges in studying the presenting symptoms and intervals to diagnosis of cancer patients, and summarize current evidence on presenting symptoms associated with a range of common and rarer cancer sites. We propose a taxonomy of cancer sites considering their symptom signature and the predictive value of common presenting symptoms. Finally, we consider evidence on associations between symptomatic presentations and intervals to diagnosis before discussing implications for the design, implementation, and evaluation of public health or health system interventions to achieve the earlier detection of cancer.

## Introduction

Diagnosing cancer earlier is a critical aim of contemporary cancer control policies. Screening interventions can achieve asymptomatic detection but are currently only available for a limited number of cancer sites, and their effectiveness is further constrained by limited sensitivity and both suboptimal and unequal uptake. This means that the majority of cancer patients continue to be diagnosed following symptomatic presentation, for whom timely diagnosis is associated with better clinical and patient-reported outcomes [1], [2], [3], [4], [5]. Diagnosing cancer at an earlier stage is also likely to be cost-effective given the increasing costs of novel drug therapies for advanced stage disease [6]. These considerations highlight the need for efforts aimed at shortening intervals to diagnosis in patients who present with symptoms.

Substantial variation in measures of diagnostic timeliness exists between patients with different cancers [7], [8], [9], [10]. Much of this variation has been attributed to the differing nature, frequency, and combinations of presenting symptoms (the ‘symptom signature’) of each cancer site (as defined in Box 1), though empirical evidence supporting this explanation is sparse. Presenting symptoms can influence the time from symptom onset to first presentation (the patient interval) and the time from first presentation to subsequent referral to specialist care (the primary care interval) [11]. Studying how different symptoms are associated with the length of these two intervals is therefore a priority for early diagnosis research.

Box 1 **Defining symptom signature and diagnostic difficulty**

In this Review, we make frequent use of two terms: **symptom signature** and **diagnostic difficulty**.

**Symptom signature** denotes the nature and relative frequency of symptoms (or symptom combinations) reported at presentation by patients later diagnosed with a particular cancer [13], [14]. We describe symptom signatures as being ‘narrow’ when most patients present with a particular symptom (as is the case for breast lump in the context of breast cancer) or ‘broad’ when patients present with a larger range of symptoms (as is the case for colorectal cancer).

The term **diagnostic difficulty** (of a given cancer site) has previously been used to characterize cancer sites as “harder-to-suspect” (e.g. multiple myeloma, pancreatic cancer) or “easier-to-suspect” (e.g. breast cancer, melanoma) based on the profile of presenting symptoms [13]. It represents the perceived predictive value for cancer of the presenting symptoms of the ‘average’ patient.

Alt-text: Box 1

We discuss methodological challenges in capturing data on symptoms at presentation and intervals to diagnosis and subsequently examine the symptom signatures of cancer sites and how this relates to diagnostic difficulty (Box 1). Diagnostic difficulty is related to the positive predictive value (PPV) of a symptom for a given disease, which is the proportion of all patients with the same symptom who will be found to have the disease. While PPV is a continuous measure, explicit threshold categories for investigation or other assessment can be considered, though until recently there have been no such applications in policy. Since 2015, the English National Institute for Health and Care Excellence (NICE) has mandated referral for specialist assessment for patients presenting in primary care with symptoms associated with a PPV for cancer that exceeds 3% [12]. This provides a practical reference point for judging the clinical significance of a symptom in the context of cancer diagnosis and has informed our interpretation throughout this Review.

Finally, we summarize available evidence on the association between symptomatic presentations and diagnostic intervals and discuss how this evidence could inform the design of early diagnosis interventions.

## How Can Presenting Symptoms and Intervals Before Diagnosis be Measured?

Capturing information on symptoms is challenging, as the majority cannot be objectively observed and their appraisal by individuals is influenced by sociocultural factors such as level of education and health literacy (including awareness of likely cancer symptoms), cancer fear, or fatalism [14], [15]. When more than one symptom is experienced, the combination of symptoms could also influence appraisal and help-seeking. Additionally, several symptoms may have conflicting or overlapping meanings in lay and professional language, and this is reflected in heterogeneous terminology in published literature. For example, abdominal bloating (uncomfortable sensation of fullness) and distension (visible increase in abdominal girth) have been used interchangeably [16], [17], while ‘change in bowel habit’ is often used by clinicians to denote a clinical suspicion of colorectal cancer beyond the presence of constipation or diarrhea alone [18]. Further, heterogeneity exists within certain nonspecific symptoms: ‘abdominal pain,’ for example, encompasses a range of presentations that vary greatly in nature, intensity, duration, and temporal evolution.

Similar challenges exist when measuring prediagnostic intervals experienced by cancer patients. Existing methods rely on the validity of the recall of particular dates of significance along the diagnostic pathway including the date of symptom onset, the date of first relevant symptomatic presentation (help-seeking), and the date of first referral to secondary care [11].

Two principal study designs have been described to examine diagnostic intervals: collecting self-reported information from patients and extracting information from patients' health records [9], [11]. We propose that these approaches are also relevant to the study of presenting symptoms (Box 2). Inconsistencies between self-reported and records-based information have been described, reflecting that both approaches have limitations [19], [20], [21], [22]. Nonetheless, medical record studies offer the opportunity to examine prediagnostic symptoms (and intervals) in large and representative samples of patients, additionally facilitating the study of patients with rarer cancers [10], [23], [24], [25].

Box 2 **Approaches to measuring presenting symptoms in cancer patient populations**

**Self-reported symptom information.**

Information on presenting symptoms can be directly elicited from patients through semistructured interviews [26], [27], [28], [29], [30], [31] or questionnaires [32], [33]. Such methods can elicit valuable first-hand insights into the symptomatic and diagnostic experience.

Patients may be prompted to identify their presenting symptoms from a predefined list (symptom recognition) or to describe them without any prompting (symptom recall), which can affect the degree of recall inaccuracies or bias. Prompting patients to consider their symptom status in respect of calendar ‘landmark’ dates (such as public holidays or events and dates of personal significance) may be helpful [34]. Studies can also be distinguished by whether the information is collected before or after the diagnosis. Collecting data about presenting symptoms after diagnosis is more convenient due to easier identification of cases but it can lead to both recall and survivorship bias. The latter results in underrepresentation of cancer patients with poor prognosis, whose presenting symptoms could be different to those of the studied patients [35]. In comparison, collecting information prospectively (before a diagnosis of cancer is made) has the advantage of minimizing such potential biases [36], [37], [38].

**Records-based symptom information.**

Alternatively, information on presenting symptoms can be recorded during healthcare encounters (e.g., with a primary care physician) and captured as part of the patients' health records [39], [40], [41]. Both coded and free-text information may be extracted [42], [43], [44], [45].

In principle, studies collecting symptom information from patient records are less prone to the risk of selection and recall bias, as information on presenting symptoms is collected prospectively and prior to diagnosis for all patients. However, such methods critically rely on the symptoms both being elicited during the consultation and being accurately recorded; in many instances, these assumptions may not be met [46], [47]. Additionally, psychosocial barriers (such as embarrassment [47], [48], [49]) and perceived or actual time pressures during the consultation [50] may prevent complete disclosure of certain symptoms to the doctor. Coded information can also be less sensitive to qualitative distinctions in symptom experience such as temporal evolution, particularly if multiple symptoms are recorded.

Alt-text: Box 2

## What are the Symptom Signatures of Different Cancer Sites?

**Figure 1:**
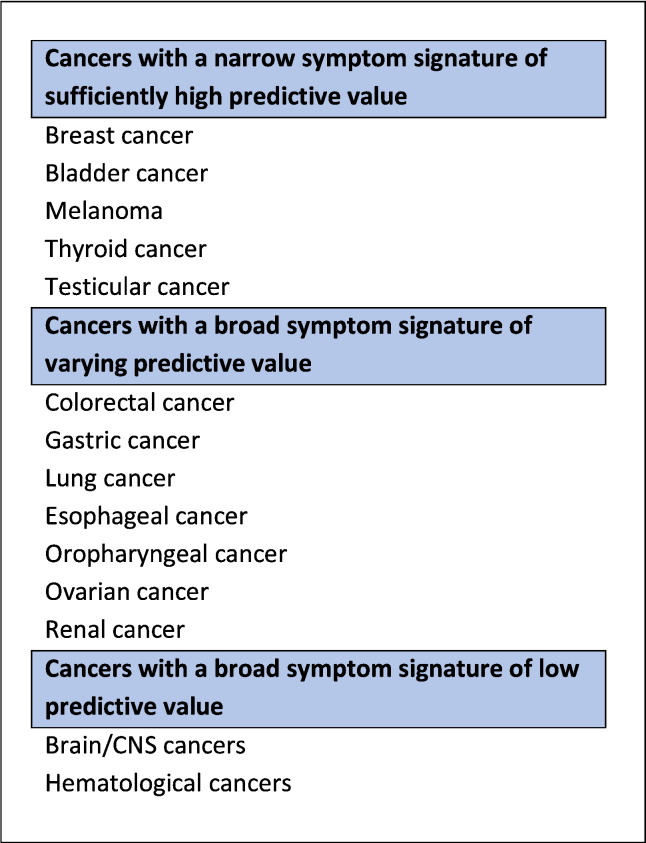
Taxonomy of cancer site–specific symptom signatures based on nature and frequency of presenting symptoms and their associated predictive value for malignancy at presentation. CNS: Central Nervous System.

Understanding the nature and relative frequency of presenting symptoms associated with different cancer sites is necessary before investigating how symptoms may influence diagnostic timeliness. We therefore reviewed the literature to examine the symptom signature of common and rarer cancers (see Box 3) and present the findings here. We consider cancers in three groups based on symptom signatures, taking into account symptom heterogeneity (the ‘breadth’ of the symptom signature) and their predictive value (see Figure 1).

Box 3 **Data sources and findings of literature review on the symptom signatures of cancer and associations between symptoms and diagnostic intervals**

**Methodology**

We searched for studies describing the frequency of presenting symptoms of cancer patients based on either primary care records or prospectively collected self-reported information, supplemented by expert knowledge of relevant evidence. Studies describing self-reported symptoms captured retrospectively (after diagnosis) were excluded due to the high risk of bias. Studies on pediatric, teenager, and young adult cancer patient populations and studies based in low- and middle-income settings were excluded as they were deemed not comparable. All retrieved studies providing evidence regarding the symptom signature of a cancer site were additionally examined for information on associations between symptoms and diagnostic intervals.

There is no standard assessment tool for risk of bias in observational nonrandomized studies, and so we developed a risk of bias tool based on the REporting of studies Conducted using Observational Routinely-collected health Data (RECORD) and the Quality Assessment of Diagnostic Accuracy Studies 2nd version (QUADAS-2) checklists [51], [52]. The resulting quality appraisal tool assessed risk of bias across six dimensions: setting, study population, symptoms, external validity, data cleaning, and other sources of bias (Table S1). This tool was used to further exclude any studies that had three or more dimensions with “high” risk of bias.

**Summary of findings**

We identified a total of 41 studies including information on presenting symptoms for 16 cancer sites (see Supplementary Material for symptom frequencies by cancer site). All included studies were based in the UK and were mostly case-control or cohort studies examining the predictive value of symptoms: for such studies, the sample size and symptom frequency relevant to cases (and not controls) were extracted. Of the included studies, 18 (44%) had low risk of bias across all examined dimensions, while 11 studies had high risk of bias across two dimensions (study population and symptom information) (Table S2). Nearly all studies focused on single cancer sites, with the exception of colorectal, esophagogastric, and renal tract cancers, which were treated as single entities, respectively [53], [54], [55], [56], [57], [58]. Most evidence related to colorectal cancer (eight studies, Table S3.4), pancreatic cancer (six studies, Table S3.9), and lung cancer (five studies, Table S3.6); only a single publication was identified for five cancers (brain, cervical, endometrial, leukemia, and myeloma).

No evidence on the frequency of presenting symptoms before diagnosis could be identified for the following 12 cancers: laryngeal cancer; liver cancer; melanoma; mesothelioma; oral cancer; penile cancer; sarcoma; small intestinal cancer; testicular cancer; thyroid cancer; vaginal cancer; and vulval cancer.

Of the 41 studies included in the Review, only four also contained evidence on associations between individual symptoms and intervals to diagnosis [36], [37], [38], [44]. One study was not included in the consideration of symptom signatures as symptom information was collected through a combination of recall and recognition; however, evidence pertaining to symptom-specific diagnostic timeliness has been considered here [22].

Alt-text: Box 3

### Cancers With a Narrow Symptom Signature

In this category, we consider several cancers where the majority of patients present with one symptom with adequately strong association with a given cancer (these are also known as ‘alarm’ symptoms). For example, the majority of women diagnosed with breast cancer initially present with a breast lump, which is associated with a relatively high predictive value for cancer; see Table 1 [44], [59], [60]. Similarly, most bladder cancer patients present with macroscopic (visible, frank) hematuria (Table S3.1) [43], [61], [62].

**Table 1:** Population-Based Estimates of the Frequencies of Presenting Symptoms Among Breast Cancer Patients [44], [59], [60]

Study	Setting/Source of Data	Study Period	Sample Size	Study Population Age/Range	Symptoms
Walker et al., 2014	Primary care, CPRD data (Read coded)	2000-09	3166	40+ years	Breast lump 44.1% Breast pain 2.4% Nipple retraction 1.0% Nipple discharge 1.0%
Redaniel et al., 2015 *	Primary care, CPRD data (Read coded)	1998-09	8544	15+ years	Breast lump 93.5% Breast pain 4.6% Nipple distortion 1.5% Nipple eczema 0.2% Breast skin changes 0.2% Bloody nipple 0.01%
Koo et al., 2017 †	Primary care, audit data (free text)	2009-10	2316	20+ years	Breast lump 83% Nipple abnormalities 6.8% Breast pain 6.4% Breast skin abnormalities 2.0% Axillary lump 1.2% Breast ulceration 1.1% Back pain 1.0% Breast contour abnormalities 0.7% Breast infection or inflammation 0.6% Breast swelling 0.6% Musculoskeletal pain 0.6% Breathlessness 0.5% Breast rash 0.4%

* All symptom frequencies calculated based on the number of breast cancer patients who had presented with a breast symptom, excluding those who were diagnosed following disclosure of family history (i.e., in the absence of any symptoms).

† Symptoms in 10 or more women listed only; further symptoms listed in Supplementary files of original paper.

Likewise, the symptomatic presentations of thyroid cancer, melanoma, testicular cancer, penile cancer, vaginal cancer, vulval cancer, and sarcoma are also narrow and are likely to have meaningfully high predictive values, although empirical documentation of the symptom signatures of these cancers is limited [63], [64]. Importantly, a relatively narrow symptom signature does not necessarily guarantee swift or easy diagnostic resolution for all patients. Firstly, the nature of the symptoms associated with sarcomas (soft tissue lump or bone pain) suggests the level of diagnostic difficulty should be low, but the relative rarity of sarcomas among the general population means that alternative diagnoses are often provided [65]. Raising awareness of these symptoms could achieve earlier diagnosis for this cancer type given the reasonable predictive values for malignancy [66], but the cost-effectiveness of a population-wide intervention for such rare cancers would be a concern. Secondly, a minority of patients with ‘narrow symptom signature’ cancers will have atypical presentations, which tend to be associated with a longer time to presentation and referral [44], [67].

### Cancers With a Broad Symptom Signature

In this category, we consider cancer sites characterized by a broad symptom signature. For some cancers, this includes certain alarm symptoms (e.g., colorectal, lung, pancreatic, esophagogastric, and ovarian cancers), while for other cancers, presenting symptoms are chiefly nonspecific (e.g., hematological malignancies, and brain and CNS cancers).

#### Broad Symptom Signature, Varying Predictive Value

Many common cancers have broad symptom signatures consisting of multiple symptoms, of which only few (e.g. one or two) are alarm symptoms that are strongly predictive of cancer. For example, eight studies report rectal bleeding, which has relatively high predictive value, as a common presenting symptom of colorectal cancer, although estimates vary substantially (16%-60%) [18], [38], [68], [69], [70], [71], [72], [73]. Other common presenting symptoms among colorectal cancer patients include abdominal pain, diarrhea, and constipation, which are associated with much lower predictive values and greater diagnostic difficulty (Table S3.4). Lung cancer also has a broad symptom signature with symptoms of varying predictive value: while it includes hemoptysis, a highly predictive symptom of malignancy [74], evidence from six studies suggests that this is a relatively rare presenting symptom, reported in less than a quarter (20%-23%) of patients subsequently diagnosed with lung cancer (Table 2) [36], [74], [75], [76].

**Table 2:** Population-Based Estimates of the Frequencies of Presenting Symptoms Among Lung Cancer Patients [36], [60], [74], [75], [76]

Study	Setting/Source of Data	Study Period	Sample Size	Study Population Age/Range	Symptoms
Hamilton et al., 2005a	Primary care, data from 21 general practices in Exeter	1998-02	247	40+ years	Hemoptysis 20% Weight loss 27% Loss of appetite 19% Dyspnea 56% Chest or rib pain 42% Fatigue 35% Finger clubbing 4.5% Thrombocytosis 14% Abnormal spirometry 9.7%
Hippisley-Cox & Coupland, 2011a	Primary care, QResearch data (Read coded)	2000-10	2196	30-84 years	Hemoptysis 23.0% *
Ades et al., 2014 †	Primary care, data from 21 general practices in Exeter	1998-02	247	40+ years	Cough 64.8% Chest pain 40.5%
Redaniel et al., 2015	Primary care, CPRD data (ead coded)	1998-09	5737	15+ years	Hemoptysis 8.8% SVC obstruction 0.4% Stridor 0.1% Anorexia 1.7% Cervical lymphadenopathy 0.5% Chest signs 2.8% Chest/rib pain 14.9% Cough 40.9% Dyspnea 18.5% Fatigue 4.1% Finger clubbing 0.5% Hoarseness 1.9% Shoulder pain 5.0%
Walter et al., 2015	Primary & secondary care data; self-reported symptoms before diagnosis	2010-12	153	40+ years	Coughing up blood 21.6% Cough or worsening cough 56.2% Breathlessness or worsening breathlessness 41.2% Chest/ shoulder pain 35.3% Hoarseness 12.4% Decreased appetite 22.2% Unexplained weight loss 15% Fatigue or tiredness 45.1% Feeling different “in yourself” 34.6%

*SVC*, superior vena cava.

* Frequencies of other symptoms included in study were not reported.

† Same study population as Hamilton et al., 2005a; frequencies of additional/different symptoms displayed only.

We identified six studies describing the frequencies of the presenting symptoms of pancreatic cancer (Table S3.9) [37], [43], [77], [78], [79], [80]. Jaundice has a high predictive value for pancreatic cancer, but reported frequencies range from 12% to 43% among patients, and it is often a sign of advanced disease [37], [43], [77], [78], [79], [80]. The most common presenting symptom among pancreatic cancer patients is abdominal pain (reported range: 40%-57% of cases), while other upper gastrointestinal symptoms such as indigestion and nausea and vomiting are also common—and given their frequency among primary care consultees, these symptoms have naturally low predictive values. Studies also reported frequencies of back pain and nonlocalizing symptoms such as weight loss, lethargy, fatigue, or malaise among considerable proportions of patients, indicating that the symptomatic picture of pancreatic cancer is usually a combination of vague and intermittent symptoms associated with considerable diagnostic difficulty (Table S3.9).

Current data on the symptom signatures of esophageal and gastric cancers are limited to studies that describe them in combination (Table S3.7) [53], [54], [55], [56]. While dysphagia is the most common presenting symptom in this cancer patient population (an alarm symptom shown to be highly predictive of malignancy), one in two patients present with a broad spectrum of other symptoms, including abdominal pain, epigastric pain, reflux, dyspepsia, and systemic symptoms such as nausea or vomiting, loss of appetite, and weight loss [53], [54], [55], [56].

Likewise, ovarian cancer has a symptom signature encompassing a broad spectrum of abdominal symptoms, although existing evidence tends to be based on smaller study populations due to the low incidence of this cancer (Table S3.8) [47], [81], [82], [83]. Abdominal distension has been shown to have a reasonable predictive value for cancer, but it is only reported by 8% to 38% of patients before diagnosis; similarly common presenting symptoms including abdominal bloating and abdominal pain have much lower predictive values [17]. Additionally, a wide variety of other symptoms with low predictive values have been identified as presenting symptoms of ovarian cancer, such as vaginal bleeding, upper and lower gastrointestinal symptoms, and systemic symptoms [47], [81], [82], [83].

#### Broad Symptom Signature, Low Predictive Value

Some cancers have broad symptom signatures comprising almost entirely symptoms of low predictive value. Results from four studies indicate that hematological cancers (leukemia, lymphoma, and multiple myeloma) have such symptom signatures, comprised of vague or nonlocalizing symptoms such as fatigue and weight loss or common complaints such as back pain (Table S13-15) [33], [84], [85], [86]. Consequently, some hematological cancers, and multiple myeloma in particular, are associated with a high level of diagnostic difficulty, as also evidenced by high frequency of multiple consultations in primary care before specialist referral [10].

Although a proportion of patients with brain cancer are diagnosed after an acute event such as a seizure or neurological deficit, most patients are thought to initially experience nonspecific symptoms, with very low predictive value (Table 3) [87]. Achieving earlier diagnosis of brain and other neurological cancers is therefore associated with substantial diagnostic difficulties.

**Table 3:** Population-Based Estimates of the Frequencies of Presenting Symptoms Among Brain or CNS Cancer Patients [87]

Study	Setting/Source of Data	Study Period	Sample Size	Study Population Age/Range	Symptoms
Hamilton et al., 2007	Primary care, CPRD data	1988-06	3505	18+ years	Headache 10.2% Motor loss 8.8% New onset seizure 4.4% Confusion 3.1% Weakness 2.7% Memory loss 1.1% Visual disorder 1.0%

## How Do Symptoms Relate to Diagnostic Intervals?

To date, there has been limited examination of individual cancer symptoms and time to diagnosis. The majority of available evidence is based on the analysis of health records, and symptoms are often aggregated into broad categories for analysis. For example, patients with alarm symptoms across a range of cancers have been shown to experience shorter diagnostic intervals (time from symptomatic presentation to diagnosis) compared to those with nonalarm symptoms [23], [60], [88], [89], and similar trends have been noted for the primary care interval among lung cancer patients [90]. Other groupings of presenting symptoms have been used among specific cancer patient populations, such as lump versus no lump among either breast or sarcoma patients [91], [92].

Available evidence on individual symptoms and diagnostic timeliness is currently limited to four cancers (breast, colorectal, lung, pancreatic) and is derived from study designs that combine prospectively collected patient information with primary and secondary care records, or examine data collected as part of clinical audit initiatives (see Box 4). Expanding this line of enquiry to other cancers is needed, with further consideration of the strengths and limitations associated with different designs.

Box 4 **Symptoms and time to diagnosis: emerging evidence**

Data from three SYMPTOM studies in England on lung, colorectal, and pancreatic cancers provide some early insights into variation in intervals to diagnosis by individual symptoms [36], [37], [38]. Symptom information was collected prospectively from patients before diagnosis and subsequently combined with information from primary and secondary care data. Investigators identified several symptoms associated with a shorter interval (e.g., chest or shoulder pain in lung cancer patients), while others were associated with longer time to diagnosis (e.g., weight loss in pancreatic cancer) [36], [37], [38]. The quantitative examination of presenting symptoms and intervals to diagnosis has also been enhanced (triangulated) with qualitative analysis of in-depth patient and healthcare professional interviews [93], [94].

The multicenter DECCIRE study used a comparable design to collect information on the diagnostic process for 795 colorectal cancer patients in Spain [95]. Symptom information was elicited from patients shortly after diagnosis by a combination of recall and recognition, and corroborated with medical records from which prediagnostic intervals were estimated [22]. Of the examined symptoms, bowel obstruction was the only independent predictor of a shorter diagnostic interval (time from symptom onset to diagnosis), although investigators noted significant differences in interval length depending on the method of data collection (patient interview, hospital records, primary care records) [22].

The presenting symptoms of breast cancer and associated patient and primary care intervals have been examined using primary care data in England [44]. The study documented that women with nonlump breast symptoms, and women with both breast lump and nonlump breast symptoms sought help later than those who presented with breast lump alone. Risks of recall or selection bias were minimal, as for the SYMPTOM/DECCIRE studies, and the study had a large representative sample (n = 2316 women) [44]. Further utilization of health records data could enable the investigation of symptom-specific timeliness of presentation and referral in greater detail.

Alt-text: Box 4

## Discussion

Measuring presenting symptoms and intervals before the diagnosis of cancer in patient populations is challenging. Currently, there are two main approaches: self-report versus records-based information. We identified 41 population-based studies describing information on the symptom signatures of 16 common and rarer cancers. Based on our findings, we described these symptom signatures as narrow (e.g., breast, bladder cancers), broad comprising cancers with some highly predictive symptoms (e.g., colorectal or pancreatic cancer), or broad comprising cancers characterised by mostly nonspecific symptoms. Evidence on how presenting symptoms relate to prediagnostic intervals was limited, but emerging findings indicate notable variation that could be used to guide interventions.

We have reviewed the interrelated concepts of symptom signature and diagnostic difficulty, the latter being an expression of symptom-specific predictive values. It should be acknowledged however that organizational or system factors can also influence the difficulty of diagnosis of a particular cancer site. For example, both the availability and the accessibility of different clinical investigations may influence the diagnostic difficulty of a cancer. Full blood counts can be more readily ordered than paraprotein studies in primary care; therefore, leukemia may be investigated with a lower threshold of cancer suspicion than myeloma and has lower overall diagnostic difficulty. Given the likely variation across countries in diagnostic activity and access to investigations, international comparisons through collaborative efforts such as the International Cancer Benchmarking Partnership (ICBP) could be valuable [96].

Further, some presenting symptoms such as jaundice are likely to represent advanced disease. In these patients, diagnostic difficulty could be minimal, but expediting their diagnosis may not necessarily lead to favorable clinical outcomes or alter prognosis. Understanding associations between symptoms and stage at diagnosis is an important area for future research [97].

There was substantial variation in reported symptom frequencies between studies, reflecting the heterogeneity in how symptom information was reported, extracted, and collated. Optimizing data capture by improving the application of existing clinical coding systems (and physician compliance) is important, particularly as novel technologies such as machine learning and natural language processing are used to extract information from electronic health records [98], [99], [100]. Until we are able to capture population-based information on symptoms systematically and reliably before diagnosis, existing methodologies such as clinical audits and prospective cohort studies offer opportunities to examine the presenting symptoms of cancer and associated diagnostic intervals [36], [37], [38], [44].

Many of the studies included in this Review investigated patients with prespecified symptoms (identified *a priori*) either from relevant literature or clinical guidelines. Rarer or less-specific symptoms might not have been captured and reported symptom frequencies may not be fully representative of the symptomatic patient population. Examining all presenting symptoms of a cohort of cancer patients without prior restrictions can bring valuable insights [36], [37], [38], [44], [101].

## Implications for Early Diagnosis Initiatives

Most patients with cancers characterized by a narrow symptom signature (such as breast and bladder cancer) experience relatively short intervals to help-seeking [7], [8], [9], [32], although a minority of patients have atypical presentations and experience prolonged intervals to presentation. Research efforts are needed to improve timely presentation in the latter group. Further, public health education campaigns about alarm symptoms remain important for improving awareness among minority groups and in the context of low- and middle-income countries [44], [102], [103], [104]. For cancers with a broad symptom signature, promoting timely help-seeking is more challenging. While many patients diagnosed with such cancers will present with alarm symptoms, many others will present with symptoms of low predictive value. Thus, while raising awareness of alarm symptoms associated with those cancers remains important, complementary strategies need to be developed. Public health education campaigns could provide information on symptom combinations or symptom duration, and also address attitudinal and psychosocial barriers to help-seeking for new symptoms, such as cancer fear and fatalism [37], [105], [106].

Postpresentation, patients with alarm symptoms are likely to benefit from fast-track diagnostic assessment pathways [24], [88], but patients with significant but nonlocalizing symptoms present a greater challenge. Innovation in diagnostic strategies, either through the development of new diagnostic tests or novel uses of existing technologies, is needed. Rapid access to specialist investigative expertise and testing strategies in the form of multidisciplinary diagnostic centers (MDCs) have recently been implemented in Denmark and are currently being developed in the United Kingdom [107], [108]. In addition to improving the diagnosis of cancer, such services can additionally improve the diagnosis of a range of other serious (nonneoplastic) diseases [107], [109], [110]. Further, for patients with nonresolving vague symptoms of low specificity, planned reevaluation through safety-netting approaches can minimize prolonged time to diagnosis [111].

In conclusion, the diagnostic difficulty of a cancer is closely tied to its symptom signature. Expanding current scientific knowledge about the nature of presenting symptoms and how they are associated with diagnostic intervals will further our understanding of mechanisms that influence the diagnostic pathway at patient, healthcare professional, and system levels. Doing so will strengthen the evidence base to support the development and implementation of public health and healthcare interventions promoting early diagnosis, thereby resulting in improved outcomes for cancer patients.

## Funding

This work was supported by a grant from the UK Department of Health [grant number no. 106/0001]. This work was part of the program of the Policy Research Unit in Cancer Awareness, Screening and Early Diagnosis. The Policy Research Unit in Cancer Awareness, Screening, and Early Diagnosis receives funding for a research program from the Department of Health Policy Research Programme. It is a collaboration between researchers from seven institutions (Queen Mary University of London, University College London, King's College London, London School of Hygiene and Tropical Medicine, Hull York Medical School, Durham University, and Peninsula Medical School/University of Exeter). G. L. is supported by a Cancer Research UK Advanced Clinician Scientist Fellowship [grant number: C18081/A18180]. F. M. W. is supported by a National Institute for Health Research (NIHR) Clinician Scientist award. The views expressed are those of the authors and not necessarily those of the Department of Health, Cancer Research UK, or NIHR.

## Appendix A Supplementary data

Supplementary material. Image 1

## References

[1] Neal RD, Tharmanathan P, France B, Din NU, Cotton S, Fallon-Ferguson J (2015). Is increased time to diagnosis and treatment in symptomatic cancer associated with poorer outcomes? Systematic review. Br J Cancer.

[2] Dahl TL, Vedsted P, Jensen H (2017). The effect of standardised cancer pathways on Danish cancer patients' dissatisfaction with waiting time. Dan Med J.

[3] Mendonca SC, Abel GA, Lyratzopoulos G (2016). Pre-referral GP consultations in patients subsequently diagnosed with rarer cancers: a study of patient-reported data. Br J Gen Pract.

[4] Robinson KM, Christensen KB, Ottesen B, Krasnik A (2012). Diagnostic delay, quality of life and patient satisfaction among women diagnosed with endometrial or ovarian cancer: a nationwide Danish study. Qual Life Res.

[5] Tørring ML, Murchie P, Hamilton W, Vedsted P, Esteva M, Lautrup M (2017). Evidence of advanced stage colorectal cancer with longer diagnostic intervals: a pooled analysis of seven primary care cohorts comprising 11 720 patients in five countries. Br J Cancer.

[6] Smith TJ, Hillner BE (2011). Bending the cost curve in cancer care. N Engl J Med.

[7] Baughan P, O'Neill B, Fletcher E (2009). Auditing the diagnosis of cancer in primary care: the experience in Scotland. Br J Cancer.

[8] Hansen RP, Vedsted P, Sokolowski I, Søndergaard J, Olesen F (2011). Time intervals from first symptom to treatment of cancer: a cohort study of 2,212 newly diagnosed cancer patients. BMC Health Serv Res.

[9] Keeble S, Abel GA, Saunders CL, McPhail S, Walter FM, Neal RD (2014). Variation in promptness of presentation among 10,297 patients subsequently diagnosed with one of 18 cancers: evidence from a National Audit of Cancer Diagnosis in Primary Care. Int J Cancer.

[10] Lyratzopoulos G, Saunders CL, Abel GA, McPhail S, Neal RD, Wardle J (2015). The relative length of the patient and the primary care interval in patients with 28 common and rarer cancers. Br J Cancer.

[11] Weller D, Vedsted P, Rubin G, Walter FM, Emery J, Scott S (2012). The Aarhus statement: improving design and reporting of studies on early cancer diagnosis. Br J Cancer.

[12] National Institute for Health and Care Excellence (2015). Suspected cancer: recognition and referral.

[13] Lyratzopoulos G, Wardle J, Rubin G (2014). Rethinking diagnostic delay in cancer: how difficult is the diagnosis?. BMJ.

[14] Smith LK, Pope C, Botha JL (2005). Patients' help-seeking experiences and delay in cancer presentation: a qualitative synthesis. Lancet.

[15] Niksic M, Rachet B, Warburton FG, Wardle J, Ramirez AJ, Forbes LJL (2015). Cancer symptom awareness and barriers to symptomatic presentation in England—are we clear on cancer?. Br J Cancer.

[16] Bankhead CR, Kehoe ST, Austoker J (2005). Symptoms associated with diagnosis of ovarian cancer: a systematic review. BJOG.

[17] Hamilton W, Peters TJ, Bankhead C, Sharp D (2009). Risk of ovarian cancer in women with symptoms in primary care: population based case-control study. BMJ.

[18] Hamilton W, Round A, Sharp D, Peters TJ (2005). Clinical features of colorectal cancer before diagnosis: a population-based case-control study. Br J Cancer.

[19] Malats N, Belloc J, Gallén M, Porta M (1995). Disagreement between hospital medical records and a structured patient interview on the type and date of the first symptom in cancers of the digestive tract. Rev Epidemiol Sante Publique.

[20] Pérez G, Porta M, Borrell C, Casamitjana M, Bonfill X, Bolibar I (2008). Interval from diagnosis to treatment onset for six major cancers in Catalonia, Spain. Cancer Detect Prev.

[21] Larsen MB, Hansen RP, Sokolowski I, Vedsted P (2014). Agreement between patient-reported and doctor-reported patient intervals and date of first symptom presentation in cancer diagnosis—a population-based questionnaire study. Cancer Epidemiol.

[22] Leiva A, Esteva M, Llobera J, Macià F, Pita-Fernández S, González-Luján L (2017). Time to diagnosis and stage of symptomatic colorectal cancer determined by three different sources of information: a population based retrospective study. Cancer Epidemiol.

[23] Din NU, Ukoumunne OC, Rubin G, Hamilton W, Carter B, Stapley S (2015). Age and gender variations in cancer diagnostic intervals in 15 cancers: analysis of data from the UK Clinical Practice Research Datalink. PLoS One.

[24] Jensen H, Torring ML, Olesen F, Overgaard J, Fenger-Gron M, Vedsted P (2015). Diagnostic intervals before and after implementation of cancer patient pathways—a GP survey and registry based comparison of three cohorts of cancer patients. BMC Cancer.

[25] Nadpara P, Madhavan SS, Tworek C (2015). Guideline-concordant timely lung cancer care and prognosis among elderly patients in the United States: a population-based study. Cancer Epidemiol.

[26] Burgess CC, Potts HWW, Hamed H, Bish AM, Hunter MS, Richards MA (2006). Why do older women delay presentation with breast cancer symptoms?. Psychooncology.

[27] Lim AW, Ramirez AJ, Hamilton W, Sasieni P, Patnick J, Forbes LJ (2014). Delays in diagnosis of young females with symptomatic cervical cancer in England: an interview-based study. Br J Gen Pract.

[28] Queenan JA, Gottlieb BH, Feldman-Stewart D, Hall SF, Irish J, Groome PA (2017). Symptom appraisal, help seeking, and lay consultancy for symptoms of head and neck cancer. Psychooncology.

[29] Walter FM, Birt L, Cavers D, Scott S, Emery J, Burrows N (2014). “This isn’t what mine looked like”: a qualitative study of symptom appraisal and help seeking in people recently diagnosed with melanoma. BMJ Open.

[30] McLachlan S, Mansell G, Sanders T, Yardley S, Van Der Windt D, Brindle L (2015). Symptom perceptions and help-seeking behaviour prior to lung and colorectal cancer diagnoses: a qualitative study. Fam Pract.

[31] Evans J, Chapple A, Salisbury H, Corrie P, Ziebland S (2014). “It can't be very important because it comes and goes”—patients' accounts of intermittent symptoms preceding a pancreatic cancer diagnosis: a qualitative study. BMJ Open.

[32] Forbes LJL, Warburton F, Richards MA, Ramirez AJ (2014). Risk factors for delay in symptomatic presentation: a survey of cancer patients. Br J Cancer.

[33] Howell DA, Warburton F, Ramirez A-J, Roman E, Smith AG, Forbes LJL (2015). Risk factors and time to symptomatic presentation in leukaemia, lymphoma and myeloma. Br J Cancer.

[34] Mills K, Emery J, Cheung C, Hall N, Birt L, Walter FM (2014). A qualitative exploration of the use of calendar landmarking instruments in cancer symptom research. BMC Fam Pract.

[35] Abel GA, Saunders CL, Lyratzopoulos G (2016). Post-sampling mortality and non-response patterns in the English Cancer Patient Experience Survey: implications for epidemiological studies based on surveys of cancer patients. Cancer Epidemiol.

[36] Walter FM, Rubin G, Bankhead C, Morris HC, Hall N, Mills K (2015). Symptoms and other factors associated with time to diagnosis and stage of lung cancer: a prospective cohort study. Br J Cancer.

[37] Walter FM, Mills K, Mendonça SC, Abel GA, Basu B, Carroll N (2016). Symptoms and patient factors associated with diagnostic intervals for pancreatic cancer (SYMPTOM pancreatic study): a prospective cohort study. Lancet Gastroenterol Hepatol.

[38] Walter FM, Emery JD, Mendonca S, Hall N, Morris HC, Mills K (2016). Symptoms and patient factors associated with longer time to diagnosis for colorectal cancer: results from a prospective cohort study. Br J Cancer.

[39] Herrett E, Gallagher AM, Bhaskaran K, Forbes H, Mathur R, van Staa T (2015). Data resource profile: Clinical Practice Research Datalink (CPRD). Int J Epidemiol.

[40] Hippisley-Cox J, Stables D, Pringle M (2004). QRESEARCH: a new general practice database for research. Inform Prim Care.

[41] Blak B, Thompson M, Dattani H, Bourke A (2011). Generalisability of The Health Improvement Network (THIN) database: demographics, chronic disease prevalence and mortality rates. J Innov Health Inform.

[42] Nadkarni PM, Ohno-Machado L, Chapman WW (2011). Natural language processing: an introduction. J Am Med Inform Assoc.

[43] Price SJ, Stapley SA, Shephard E, Barraclough K, Hamilton WT (2016). Is omission of free text records a possible source of data loss and bias in Clinical Practice Research Datalink studies? A case-control study. BMJ Open.

[44] Koo MM, von Wagner C, Abel G, McPhail S, Rubin GP, Lyratzopoulos G (2017). Typical and atypical symptoms in women with breast cancer: evidence of variation in diagnostic intervals from a national audit of cancer diagnosis. Cancer Epidemiol.

[45] National Academies of Sciences, Engineering, and Medicine (2015). Improving Diagnosis in Health Care.

[46] Spechler SJ, Jain SK, Tendler DA, Parker RA (2002). Racial differences in the frequency of symptoms and complications of gastro-oesophageal reflux disease. Aliment Pharmacol Ther.

[47] Lim A, Mesher D, Gentry-Maharaj A, Balogun N, Widschwendter M, Jacobs I (2016). Time to diagnosis of type I or II invasive epithelial ovarian cancers: a multicentre observational study using patient questionnaire and primary care records. BJOG.

[48] Forbes LJL, Simon AE, Warburton F, Boniface D, Brain KE, Dessaix A (2013). Differences in cancer awareness and beliefs between Australia, Canada, Denmark, Norway, Sweden and the UK (the International Cancer Benchmarking Partnership): do they contribute to differences in cancer survival?. Br J Cancer.

[49] Whitaker KL, Macleod U, Winstanley K, Scott SE, Wardle J (2015). Help seeking for cancer “alarm” symptoms: a qualitative interview study of primary care patients in the UK. Br J Gen Pract.

[50] Cromme SK, Whitaker KL, Winstanley K, Renzi C, Smith CF, Wardle J (2016). Worrying about wasting GP time as a barrier to help-seeking: a community-based, qualitative study. Br J Gen Pract.

[51] Whiting PF (2011). QUADAS-2: a revised tool for the quality assessment of diagnostic accuracy studies. Ann Intern Med.

[52] Benchimol EI, Smeeth L, Guttmann A, Harron K, Moher D, Petersen I (2015). The REporting of studies Conducted using Observational Routinely-collected health Data (RECORD) statement. PLoS Med.

[53] Stephens MR, Lewis WG, White S, Blackshaw GRJC, Edwards P, Barry JD (2005). Prognostic significance of alarm symptoms in patients with gastric cancer. Br J Surg.

[54] Hippisley-Cox J, Coupland C (2011). Identifying patients with suspected gastro-oesophageal cancer in primary care: derivation and validation of an algorithm. Br J Gen Pract.

[55] Collins GS, Altman DG (2013). Identifying patients with undetected gastro-oesophageal cancer in primary care: external validation of QCancer® (Gastro-Oesophageal). Eur J Cancer.

[56] Stapley S, Peters TJ, Neal RD, Rose PW, Walter FM, Hamilton W (2013). The risk of oesophago-gastric cancer in symptomatic patients in primary care: a large case-control study using electronic records. Br J Cancer.

[57] Hippisley-Cox J, Coupland C. Identifying patients with suspected renal tract cancer in primary care: derivation and validation of an algorithm 2012:251–60. https://doi.org/10.3399/bjgp12X636074.e251. 10.3399/bjgp12X636074 PMC3310031 22520912

[58] Collins GS, Altman DG (2013). Identifying patients with undetected renal tract cancer in primary care: an independent and external validation of QCancer® (Renal) prediction model. Cancer Epidemiol.

[59] Walker S, Hyde C, Hamilton W (2014). Risk of breast cancer in symptomatic women in primary care: a case-control study using electronic records. Br J Gen Pract.

[60] Redaniel MT, Martin RM, Ridd MJ, Wade J, Jeffreys M (2015). Diagnostic intervals and its association with breast, prostate, lung and colorectal cancer survival in England: historical cohort study using the Clinical Practice Research Datalink. PLoS One.

[61] Shephard EA, Stapley S, Neal RD, Rose P, Walter FM, Hamilton WT (2012). Clinical features of bladder cancer in primary care. Br J Gen Pract.

[62] Price SJ, Shephard EA, Stapley SA, Barraclough K, Hamilton WT (2014). Non-visible versus visible haematuria and bladder cancer risk: a study of electronic records in primary care. Br J Gen Pract.

[63] Avilés-Izquierdo JA, Molina-López I, Rodríguez-Lomba E, Marquez-Rodas I, Suarez-Fernandez R, Lazaro-Ochaita P (2016). Who detects melanoma? Impact of detection patterns on characteristics and prognosis of patients with melanoma. J Am Acad Dermatol.

[64] Kobayashi K, Saito T, Kitamura Y, Nobushita T, Kawasaki T, Hara N (2014). Effect of the time from the presentation of symptoms to medical consultation on primary tumor size and survival in patients with testicular cancer: Shift in the last 2 decades. Urol Oncol Semin Orig Investig.

[65] Smith GM, Johnson GD, Grimer RJ, Wilson S (2011). Trends in presentation of bone and soft tissue sarcomas over 25 years: little evidence of earlier diagnosis. Ann R Coll Surg Engl.

[66] Dyrop HB, Vedsted P, Safwat A, Maretty-Nielsen K, Hansen BH, Jørgensen PH (2014). Alarm symptoms of soft-tissue and bone sarcoma in patients referred to a specialist center. Acta Orthop.

[67] Schmidt-Hansen M, Berendse S, Hamilton W (2015). The association between symptoms and bladder or renal tract cancer in primary care: a systematic review. Br J Gen Pract.

[68] Stapley S, Peters TJ, Sharp D, Hamilton W (2006). The mortality of colorectal cancer in relation to the initial symptom at presentation to primary care and to the duration of symptoms: a cohort study using medical records. Br J Cancer.

[69] Esteva M, Leiva A, Ramos M, Pita-Fernández S, González-Luján L, Casamitjana M (2013). Factors related with symptom duration until diagnosis and treatment of symptomatic colorectal cancer. BMC Cancer.

[70] Hamilton W, Lancashire R, Sharp D, Peters TJ, Cheng K, Marshall T (2009). The risk of colorectal cancer with symptoms at different ages and between the sexes: a case-control study. BMC Med.

[71] Collins GS, Altman DG (2012). Identifying patients with undetected colorectal cancer: an independent validation of QCancer (Colorectal). Br J Cancer.

[72] Hippisley-Cox J, Coupland C (2012). Identifying patients with suspected colorectal cancer in primary care: derivation and validation of an algorithm. Br J Gen Pract.

[73] Renzi C, Lyratzopoulos G, Card T, Chu TPC, Macleod U, Rachet B (2016). Do colorectal cancer patients diagnosed as an emergency differ from non-emergency patients in their consultation patterns and symptoms? A longitudinal data-linkage study in England. Br J Cancer.

[74] Hamilton W, Peters TJ, Round A, Sharp D (2005). What are the clinical features of lung cancer before the diagnosis is made? A population based case-control study. Thorax.

[75] Ades AE, Biswas M, Welton NJ, Hamilton W (2014). Symptom lead time distribution in lung cancer: natural history and prospects for early diagnosis. Int J Epidemiol.

[76] Hippisley-Cox J, Coupland C (2011). Identifying patients with suspected lung cancer in primary care: derivation and validation of an algorithm. Br J Gen Pract.

[77] Stapley S, Peters TJ, Neal RD, Rose PW, Walter FM, Hamilton W (2012). The risk of pancreatic cancer in symptomatic patients in primary care: a large case-control study using electronic records. Br J Cancer.

[78] Hippisley-Cox J, Coupland C (2012). Identifying patients with suspected pancreatic cancer in primary care: derivation and validation of an algorithm. Br J Gen Pract.

[79] Collins GS, Altman DG (2013). Identifying patients with undetected pancreatic cancer in primary care: an independent and external validation of QCancer® (Pancreas). Br J Gen Pract.

[80] Keane MG, Horsfall L, Rait G, Pereira SP (2014). A case-control study comparing the incidence of early symptoms in pancreatic and biliary tract cancer. BMJ Open.

[81] Hamilton W (2009). Five misconceptions in cancer diagnosis. Br J Gen Pract.

[82] Hippisley-Cox J, Coupland C (2011). Identifying women with suspected ovarian cancer in primary care: derivation and validation of algorithm. BMJ.

[83] Collins GS, Altman DG (2013). Identifying women with undetected ovarian cancer: independent and external validation of QCancer ® (Ovarian) prediction model. Eur J Cancer Care (Engl).

[84] Shephard EA, Neal RD, Rose PW, Walter FM, Hamilton W (2016). Symptoms of adult chronic and acute leukaemia before diagnosis: large primary care case-control studies using electronic records. Br J Gen Pract.

[85] Shephard EA, Neal RD, Rose PW, Walter FM, Hamilton WT (2015). Quantifying the risk of Hodgkin lymphoma in symptomatic primary care patients aged ≥40 years: a case-control study using electronic records. Br J Gen Pract.

[86] Shephard EA, Neal RD, Rose P, Walter FM, Litt EJ, Hamilton WT (2015). Quantifying the risk of multiple myeloma from symptoms reported in primary care patients: a large case-control study using electronic records. Br J Gen Pract.

[87] Hamilton W, Kernick D (2007). Clinical features of primary brain tumours: a case-control study using electronic primary care records. Br J Gen Pract.

[88] Neal RD, Din NU, Hamilton W, Ukoumunne OC, Carter B, Stapley S (2014). Comparison of cancer diagnostic intervals before and after implementation of NICE guidelines: analysis of data from the UK General Practice Research Database. Br J Cancer.

[89] Jensen H, Tørring ML, Olesen F, Overgaard J, Vedsted P (2014). Cancer suspicion in general practice, urgent referral and time to diagnosis: a population-based GP survey and registry study. BMC Cancer.

[90] Guldbrandt L, Fenger-Grøn M, Rasmussen T, Jensen H, Vedsted P (2015). The role of general practice in routes to diagnosis of lung cancer in Denmark: a population-based study of general practice involvement, diagnostic activity and diagnostic intervals. BMC Health Serv Res.

[91] Webber C, Jiang L, Grunfeld E, Groome PA (2017). Identifying predictors of delayed diagnoses in symptomatic breast cancer: a scoping review. Eur J Cancer Care (Engl).

[92] Dyrop HB, Safwat A, Vedsted P, Maretty-Kongstad K, Hansen BH, Jørgensen PH (2016). Characteristics of 64 sarcoma patients referred to a sarcoma center after unplanned excision. J Surg Oncol.

[93] Hall N, Birt L, Banks J, Emery J. Symptom appraisal and healthcare-seeking for symptoms suggestive of colorectal cancer: a qualitative study 2015;5:e008448. doi:https://doi.org/10.1136/bmjopen-2015-008448. 10.1136/bmjopen-2015-008448 PMC4606388 26453591

[94] Mills K, Birt L, Emery JD, Hall N, Banks J, Johnson M (2017). Understanding symptom appraisal and help-seeking in people with symptoms suggestive of pancreatic cancer: a qualitative study. BMJ Open.

[95] Esteva M, Ramos M, Cabeza E, Llobera J, Ruiz A, Pita S (2007). Factors influencing delay in the diagnosis of colorectal cancer: a study protocol. BMC Cancer.

[96] Butler J, Foot C, Bomb M, Hiom S, Coleman M, Bryant H (2013). The International Cancer Benchmarking Partnership: an international collaboration to inform cancer policy in Australia, Canada, Denmark, Norway, Sweden and the United Kingdom. Health Policy (NY).

[97] Ewing M, Naredi P, Zhang C, Mansson J (2016). Identification of patients with non-metastatic colorectal cancer in primary care: a case-control study. Br J Gen Pract.

[98] Afzal Z, Schuemie MJ, van Blijderveen JC, Sen EF, Sturkenboom MC, Kors JA (2013). Improving sensitivity of machine learning methods for automated case identification from free-text electronic medical records. BMC Med Inform Decis Mak.

[99] Rumball-Smith J, Shekelle PG, Bates DW (2017). Using the electronic health record to understand and minimize overuse. JAMA.

[100] Kostopoulou O, Porat T, Corrigan D, Mahmoud S, Delaney BC (2017). Diagnostic accuracy of GPs when using an early-intervention decision support system: a high-fidelity simulation. Br J Gen Pract.

[101] Bailey SER, Ukoumunne OC, Shephard EA, Hamilton W (2016). Clinical relevance of thrombocytosis in primary care: a prospective cohort study of cancer incidence using UK electronic medical records and cancer registry data. Br J Gen Pract.

[102] Anderson BO, Cazap E, El Saghir NS, Yip C-H, Khaled HM, Otero IV (2011). Optimisation of breast cancer management in low-resource and middle-resource countries: executive summary of the Breast Health Global Initiative consensus, 2010. Lancet Oncol.

[103] Jones CE, Maben J, Jack RH, Davies EA, Forbes LJ, Lucas G (2014). A systematic review of barriers to early presentation and diagnosis with breast cancer among black women. BMJ Open.

[104] Youl P, Aitken J, Turrell G, Chambers S, Dunn J, Pyke C (2016). The impact of rurality and disadvantage on the diagnostic interval for breast cancer in a large population-based study of 3202 women in Queensland, Australia. Int J Environ Res Public Health.

[105] Winstanley K, Renzi C, Smith CF, Wardle J, Whitaker KL (2016). The impact of body vigilance on help-seeking for cancer “alarm” symptoms: a community-based survey. BMC Public Health.

[106] Fish JA, Prichard I, Ettridge K, Grunfeld EA, Wilson C (2015). Psychosocial factors that influence men's help-seeking for cancer symptoms: a systematic synthesis of mixed methods research. Psychooncology.

[107] Fuller E, Fitzgerald K, Hiom S (2016). Accelerate, Coordinate, Evaluate Programme: a new approach to cancer diagnosis. Br J Gen Pract.

[108] Vedsted P, Olesen F (2015). A differentiated approach to referrals from general practice to support early cancer diagnosis—the Danish three-legged strategy. Br J Cancer.

[109] Moseholm E, Lindhardt B (2017). Patient characteristics and cancer prevalence in the Danish cancer patient pathway for patients with serious non-specific symptoms and signs of cancer—a nationwide, population-based cohort study. Cancer Epidemiol.

[110] Næser E, Fredberg U, Møller H, Vedsted P (2017). Clinical characteristics and risk of serious disease in patients referred to a diagnostic centre: a cohort study. Cancer Epidemiol.

[111] Nicholson BD, Mant D, Bankhead C (2016). Can safety-netting improve cancer detection in patients with vague symptoms?. BMJ.

