# Supplementary material for: Symptom Signatures and Diagnostic Timeliness in Cancer Patients: A Review of Current Evidence
Source: Neoplasia. 2017 Dec 16;20(2):165–74. doi: 10.1016/j.neo.2017.11.005 (PMC5735300; doi:10.1016/j.neo.2017.11.005)
Supplement: Supplementary file 1 — Supplementary material. [file mmc1.docx]

# Supplementary Material

**Supplementary table S1 ­**– Risk of bias tool for observational non-randomized studies

**Supplementary table S2** – Assessment of risk of bias in included studies

**Supplementary table S3.1-15** – Available evidence on cancer symptom signatures

# Supplementary table S1

## Risk of bias tool for observational non-randomized studies

The following risk of bias tool is based on the REporting of studies Conducted using Observational Routinely-collected health Data (RECORD) and Quality Assessment of Diagnostic Accuracy Studies 2^nd^ version (QUADAS-2) checklists [1,2]. The risk of bias is assessed as ‘low’, ‘high’, or ‘unclear’ across six dimensions: setting; study population; symptoms; external validity; data cleaning; and other sources of bias (see below).

| **Section of paper** | **Criteria to judge (bold print indicates summary of section)** | **Comments (Low/ High/ Unclear)** |
| --- | --- | --- |
| Methods – setting | 5. Describe the setting, locations, and relevant dates, including periods of recruitment, exposure, follow-up, and data collection  ***In other words, could the setting of the study have introduced bias?*** |  |
| Methods – Participants | 6. (a) Cohort study - Give the eligibility criteria, and the sources and methods of selection of participants. Describe methods of follow-up  Case-control study - Give the eligibility criteria, and the sources and methods of case ascertainment and control selection. Give the rationale for the choice of cases and controls  Cross-sectional study - Give the eligibility criteria, and the sources and methods of selection of participants  ----------------------------------------------------------------  6. (b) Cohort study - For matched studies, give matching criteria and number of exposed and unexposed  Case-control study - For matched studies, give matching criteria and the number of controls per case  6.1: The methods of study population selection (such as codes or algorithms used to identify subjects) should be listed in detail. If this is not possible, an explanation should be provided.  6.2: Any validation studies of the codes or algorithms used to select the population should be referenced. If validation was conducted for this study and not published elsewhere, detailed methods and results should be provided.  ----------------------------------------------------------------  Domain 1: patient selection  A. Risk of bias  1A1: Was a consecutive or random sample of patients enrolled? (Yes/No/Unclear)  1A3: Did the study avoid inappropriate exclusions? (Yes/No/Unclear)  ***Could the selection of patients have introduced bias? Also see Results section*** |  |
| Data sources/ measurement | 8. For each variable of interest, give sources of data and details of methods of assessment (measurement).  Describe comparability of assessment methods if there is more than one group  ***Could the measurement of symptoms have introduced bias?*** |  |
| Study size | 10. Explain how the study size was arrived at  ***Could the study size have affected the external validity of results? Also see generalisability*** |  |
| Data access and cleaning methods | 12.1: Authors should describe the extent to which the investigators had access to the database population used to create the study population.  12.2: Authors should provide information on the data cleaning methods used in the study.  ***Could data cleaning have introduced bias?*** |  |
| Results – Participants | 13. (a) Report the numbers of individuals at each stage of the study (e.g., numbers potentially eligible, examined for eligibility, confirmed eligible, included in the study, completing follow-up, and analysed)  13. (b) Give reasons for non-participation at each stage.  13. (c) Consider use of a flow diagram  ----------------------------------------------------------------  13.1: Describe in detail the selection of the persons included in the study (i.e., study population selection) including filtering based on data quality, data availability and linkage. The selection of included persons can be described in the text and/or by means of the study flow diagram.  ***Could the selection of patients have introduced bias? Also see Methods section*** |  |
| Discussion | 19. Discuss limitations of the study, taking into account sources of potential bias or imprecision. Discuss both direction and magnitude of any potential bias  ----------------------------------------------------------------  19.1: Discuss the implications of using data that were not created or collected to answer the specific research question(s). Include discussion of misclassification bias, unmeasured confounding, missing data, and changing eligibility over time, as they pertain to the study being reported.  ***Were there other potential sources of bias mentioned by the authors?*** |  |
| Generalisability | 21. Discuss the generalisability (external validity) of the study results  ***Could the study size have affected the external validity of results? Also see study size*** |  |

# Supplementary table S2

## Assessment of risk of bias in included studies

Studies included in the Review had low risk of bias for at least three of the six dimensions.

|  |  |  | **Dimensions of risk of bias†** | | | | | |
| --- | --- | --- | --- | --- | --- | --- | --- | --- |
| **Cancer** | **Paper** | **Sample size** | **Setting?** | **Study population?** | **Symptoms?** | **External validity?** | **Data cleaning?** | **Other sources of bias?** |
| Bladder* | Shephard et al., 2012 | 4915 | + | + | + | + | + | + |
|  | Price et al., 2014 | 4915 | + | + | + | + | + | + |
|  | Price et al., 2016^1^ | 4915 | + | + | – | + | + | + |
| Brain | Hamilton et al., 2007 | 3505 | + | + | + | + | + | + |
| Breast | Walker et al., 2014 | 3166 | + | + | + | + | + | + |
|  | Redaniel et al., 2015^2^ | 8544 | + | + | ? | + | ? | + |
|  | Koo et al., 2017 | 2316 | + | + | + | + | + | + |
| Cervical | Walker et al., 2017 | 885 | + | + | + | + | + | + |
| Colorectal | Hamilton et al., 2005a | 349 | + | + | + | + | + | + |
|  | Stapley et al., 2006 | 349 | + | + | – | + | + | + |
|  | Hamilton et al., 2009a | 5477 | + | + | + | + | ? | + |
|  | Hippisley-Cox & Coupland 2012a | 2603 | + | – | – | + | + | + |
|  | Collins & Altman 2012a | 3712 | + | – | – | + | + | + |
|  | Redaniel et al., 2015^2^ | 5912 | + | + | ? | + | ? | + |
|  | Walter et al., 2016a | 152 | + | – | + | + | + | + |
|  | Renzi et al., 2016 | 1606 | + | + | + | + | + | + |
| Endometrial | Walker et al., 2013 | 3166 | + | + | + | + | + | + |
| Leukemia | Shephard et al., 2016^3^ | 3814 | + | + | – | + | + | + |
| Lung | Hamilton et al., 2005b | 247 | + | + | + | + | + | + |
|  | Hippisley-Cox & Coupland, 2011a | 2196 | + | – | – | + | + | + |
|  | Ades et al., 2014 | 247 | + | + | + | + | ? | + |
|  | Redaniel et al., 2015^2^ | 5737 | + | + | ? | + | ? | + |
|  | Walter et al., 2015 | 153 | + | – | + | + | + | + |
| Lymphoma | Shephard et al., 2015a | 283 | + | + | + | + | + | + |
|  | Shephard et al., 2015b | 4362 | + | + | + | + | + | + |
| Myeloma | Shephard et al., 2015c | 2703 | + | + | + | + | + | + |
| Esophago-gastric | Stephens et al., 2005 | 300 | + | – | + | + | + | + |
|  | Hippisley-Cox & Coupland 2011b | 781 | + | – | – | + | + | + |
|  | Collins & Altman 2012b | 287 | + | – | – | + | + | + |
|  | Stapley et al., 2013 | 7471 | + | + | + | + | + | + |
| Ovarian | Hamilton et al., 2009b | 212 | + | + | + | + | + | + |
|  | Hippisley-Cox & Coupland 2012b | 538 | + | – | – | + | + | + |
|  | Collins & Altman 2012c | 735 | + | – | – | + | + | + |
|  | Lim et al., 2015 | 182 | + | + | + | + | + | + |
| Pancreatic | Stapley et al., 2012 | 3635 | + | + | + | + | + | + |
|  | Hippisley-Cox & Coupland 2012c | 781 | + | – | – | + | + | + |
|  | Collins & Altman 2013a | 287 | + | – | – | + | + | + |
|  | Keane et al., 2014 | 296 | + | + | + | + | + | + |
|  | Walter et al., 2016b | 391 | + | – | + | + | + | + |
|  | Price et al., 2016^1^ | 561 | + | + | – | + | + | + |
| Prostate | Hamilton et al., 2006 | 217 | + | + | + | + | + | + |
|  | Redaniel et al., 2015^2^ | 1763 | + | + | ? | + | ? | + |
| Renal | Shephard et al., 2013 | 3149 | + | + | + | + | + | + |
|  | Hippisley-Cox & Coupland 2012d^4^ | 1622 | + | – | – | + | + | + |
|  | Collins & Altman 2013b^4^ | 2283 | + | – | – | + | + | + |

† ‘+’ denotes low risk of bias; ‘?’ denotes unclear risk of bias; ‘-‘ denotes high risk of bias

*the three studies examine the same patient population (n=4915) but with different methodologies

^1^ includes independent symptom frequencies for bladder cancer and pancreatic cancer patients

^2^ includes independent symptom frequencies for breast, colorectal, lung, and prostate cancer patients

^2^ includes patients with acute leukemia (n=937) + chronic leukemia (n=2877)

^4^ reported information on renal tract cancer patients (including bladder cancer)

# Supplementary table S3

## Cancers with a narrow symptom signature

#### S3.1 Bladder cancer

| Table 1 Population-based estimates of the frequencies of presenting symptoms among bladder cancer patients [3–5] | | | | | |
| --- | --- | --- | --- | --- | --- |
| **Study** | **Setting/ source of data** | **Study period** | **Sample size** | **Study population age/range** | **Symptoms** |
| Shephard et al., 2012 | Primary care, CPRD data (Read coded) | 2000–09 | 4915 | 40+ years | Visible hematuria 53% Invisible hematuria 2.6%^1^ Dysuria 9% Abdominal pain 7% Constipation 6% Urinary tract infection 17% |
| Price et al., 2014 ^2^ | Primary care, CPRD data (Read coded & uncoded data) | 2000–09 | 4915 | 40+ years | Macroscopic hematuria 64.0% Microscopic hematuria 6.4% |
| Price et al., 2016 ^3^ | Primary care, CPRD data (Read coded & uncoded data) | 2000–09 | 4935 | 40+ years | Visible hematuria 63.8% Abdominal pain 12.2%  Jaundice 0.4% |

^1^ as reported by Price et al., 2014

^2^ same study population as Shephard et al., 2012 & Price et al., 2016 but used uncoded data to examine hematuria only

^3^ majority of patients derived from same study population as Shephard et al., 2012 & Price et al., 2014 but represents a slightly different analysis encompassing purposefully selected symptoms.

#### S3.2 Breast cancer

| Table 2 Population-based estimates of the frequencies of presenting symptoms among breast cancer patients [6–8] | | | | | |
| --- | --- | --- | --- | --- | --- |
| **Study** | **Setting/ source of data** | **Study period** | **Sample size** | **Study population age/range** | **Symptoms** |
| Walker et al., 2014 | Primary care, CPRD data (Read coded) | 2000–09 | 3166 | 40+ years | Breast lump 44.1% Breast pain 2.4% Nipple retraction 1.0%  Nipple discharge 1.0% |
| Redaniel et al., 2015 ^1^ | Primary care, CPRD data (Read coded) | 1998–09 | 8544 | 15+ years | Breast lump 93.5%  Breast pain 4.6%  Nipple distortion 1.5%  Nipple eczema 0.2%  Breast skin changes 0.2%  Bloody nipple 0.01% |
| Koo et al., 2017 ^2^ | Primary care, audit data (free-text) | 2009–10 | 2316 | 20+ years | Breast lump 83%  Nipple abnormalities 6.8%  Breast pain 6.4%  Breast skin abnormalities 2.0%  Axillary lump 1.2%  Breast ulceration 1.1%  Back pain 1.0%  Breast contour abnormalities 0.7%  Breast infection or inflammation 0.6%  Breast swelling 0.6%  Musculoskeletal pain 0.6%  Breathlessness 0.5%  Breast rash 0.4% |

^1^ All symptom frequencies calculated manually based on the number of breast cancer patients who had presented with a breast symptom, excluding those who were diagnosed following disclosure of family history (i.e. in the absence of any symptoms)

^2^ Symptoms in 10 or more women listed only; further symptoms listed in Supplementary files of original paper

## Cancers with a broad symptom signature, varying predictive value

#### S3.3 Cervical cancer

| Table 3 Population-based estimates of the frequencies of presenting symptoms among cervical cancer patients [9] | | | | | |
| --- | --- | --- | --- | --- | --- |
| **Study** | **Setting/ source of data** | **Study period** | **Sample size** | **Study population age/range** | **Symptoms** |
| Walker et al., 2017 | Primary care, CPRD data (Read coded) | 2000–09 | 885 | 40+ years | Post-menopausal bleeding 20.7%  Abdominal pain 8.1%  Vaginal discharge or vaginitis 7.7%  Urinary tract infection 7.6%  Hematuria 2.7%  Irregular menstruation 2.3%  Inter-menstrual bleeding 1.2% |

#### S3.4 Colorectal cancer

| Table 4 Population-based estimates of the frequencies of presenting symptoms among colorectal cancer patients [8,10–16] | | | | | |
| --- | --- | --- | --- | --- | --- |
| **Study** | **Setting/ source of data** | **Study period** | **Sample size** | **Study population age/range** | **Symptoms** |
| Hamilton et al., 2005a | Primary care, data from 21 general practices in Exeter | 1998–02 | 349 | 40+ years | Rectal bleeding 42.4% Abdominal pain 42.4% Diarrhea 37.8% Constipation 26.1%  Weight loss 26.9% |
| Stapley et al., 2006 ^1^ | Primary care, data from 21 general practices in Exeter | 1998–02 | 349 | 40+ years | Rectal bleeding 39% Abdominal pain 38% Diarrhea 32% Constipation 23%  Weight loss 23% |
| Hamilton et al., 2009a | Primary care,  THIN data (Read coded) | 2001–06 | 5477 | 30+ years | Abdominal pain 29.7%  Constipation 27.0% Diarrhea 18.0% Rectal bleeding 15.6%  Change in bowel habit 11.2%  Weight loss 10.2% ^2^ |
| Hippisley-Cox & Coupland, 2012a | Primary care,  QResearch data (Read coded) | 2000–10 | 2603 | 30–84 years | Rectal bleeding 32.3%  Abdominal pain 32.5%  Appetite loss 1.8%  Weight loss 4.1%  Change in bowel habit 1.5% ^3^ |
| Collins & Altman, 2012a ^4^ | Primary care,  THIN data (Read coded) | 2000–08 | 3712 | 30–84 years | Rectal bleeding 36.7%  Abdominal pain 32.9%  Appetite loss 1.2%  Weight loss 5.8%  Change in bowel habit 2.4% ^3^ |
| Redaniel et al., 2015 | Primary care, CPRD data (Read coded) | 1998–09 | 5912 | 15+ years | Abdominal pain 28%  Anorexia 0.8%  Constipation 11.5%  Diarrhea 14.9%  Fatigue 4.6%  Weight loss 3.5% |
| Walter et al., 2016a ^5^ | Primary & secondary care data; self-reported symptoms before diagnosis | 2010–12 | 152 | 40+ years | Change in bowel habit 61%  Rectal bleeding 60%  Indigestion, reflux, or persistent abdominal pain 28%  Fatigue or tiredness 38%  Feeling different “in yourself” 25%  Loss of appetite 20%  Back pain 8%  Weight loss 16%  Acute gastro-intestinal (GI) illness 2%  Perianal pain or discomfort 2%  Wind or flatulence 1%  Urgency or leakage of bowels 0%  Bloating 1%  Mucus or discharge per rectum 1%  Non-abdominal pain 1% |
| Renzi et al., 2016 ^6^ | Primary care,  CPRD data  (Read coded) | 2005–06 | 1606 | 25+ years | Abdominal pain 31.1%  Rectal bleeding 22.7% Anemia 19.6%  Diarrhea 15.5%  Change in bowel habit 12.8%  Constipation 12.6%  Fatigue 4.5%  Weight loss 4.4% |

^1^ same study population as Hamilton et al., 2005b but included presenting symptoms from 1 year before diagnosis only

^2^ calculated by combining frequencies of two weight loss categories (5–10% weight loss, ≥10% weight loss)

^3^ frequency of change in bowel habit was calculable among male patients only

^4^ all symptom frequencies calculated manually based on published data; frequencies based on symptoms reported separately for men and women diagnosed with colorectal cancer

^5^ all symptom frequencies calculated manually based on published data by combining frequencies of colorectal cancer patients who experienced symptom as a “first symptom” or “subsequent symptom”

^6^ all symptom frequencies calculated manually based on published data by combining frequencies of symptoms that occurred 1 year before diagnosis among colon (n=1029) and rectal (n=577) cancer patients

#### S3.5 Endometrial cancer

| Table 5 Population-based estimates of the frequencies of presenting symptoms among endometrial cancer patients [17] | | | | | |
| --- | --- | --- | --- | --- | --- |
| **Study** | **Setting/ source of data** | **Study period** | **Sample size** | **Study population age/range** | **Symptoms** |
| Walker et al., 2013 | Primary care, CPRD data (Read coded) | 2000–09 | 3166 | 40+ years | General abnormal vaginal bleeding 63%  Post-menopausal bleeding ^1^ 33.9%  Post-menopausal bleeding ^2^ 12.8%  Excessive bleeding 4.0%  Irregular menstruation ^1^ 15.6%  Irregular menstruation ^2^ 5.6%  Vaginal discharge 7.5%  Hematuria 4.4%  Abdominal pain ^1^ 5.1%  Abdominal pain ^2^ 2.3% |

^1^ frequency of symptom reported on one visit before diagnosis

^2^ frequency of symptom reported on two or more visits before diagnosis

#### S3.6 Lung cancer

| Table 6 Population-based estimates of the frequencies of presenting symptoms among lung cancer patients [18,8,19–21] | | | | | |
| --- | --- | --- | --- | --- | --- |
| **Study** | **Setting/ source of data** | **Study period** | **Sample size** | **Study population age/range** | **Symptoms** |
| Hamilton et al., 2005b | Primary care, data from 21 general practices in Exeter | 1998–02 | 247 | 40+ years | Hemoptysis 20% Weight loss 27% Loss of appetite 19% Dyspnea 56% Chest or rib pain 42% Fatigue 35% Finger clubbing 4.5% Thrombocytosis 14% Abnormal spirometry 9.7% |
| Hippisley-Cox & Coupland, 2011a | Primary care,  QResearch data (Read coded) | 2000–10 | 2196 | 30–84 years | Hemoptysis 23.0% ^1^ |
| Ades et al., 2014 ^2^ | Primary care, data from 21 general practices in Exeter | 1998–02 | 247 | 40+ years | Cough 64.8%  Chest pain 40.5% |
| Redaniel et al., 2015 | Primary care, CPRD data (Read coded) | 1998–09 | 5737 | 15+ years | Hemoptysis 8.8%  SVC obstruction 0.4%  Stridor 0.1%  Anorexia 1.7%  Cervical lymphadenopathy 0.5%  Chest signs 2.8%  Chest/rib pain 14.9%  Cough 40.9%  Dyspnea 18.5%  Fatigue 4.1%  Finger clubbing 0.5%  Hoarseness 1.9%  Shoulder pain 5.0% |
| Walter et al., 2015 | Primary & secondary care data; self-reported symptoms before diagnosis | 2010–12 | 153 | 40+ years | Coughing up blood 21.6% Cough or worsening cough >3 weeks 56.2% Breathlessness or worsening breathlessness 41.2% Chest/ shoulder pain 35.3% Hoarseness 12.4% Decreased appetite 22.2% Unexplained weight loss 15% Fatigue or tiredness 45.1% Feeling different “in yourself” 34.6% |

SVC: superior vena cava

^1^ Frequencies of other symptoms included in study were not reported

^2^ Same study population as Hamilton et al., 2005a; frequencies of additional/different symptoms displayed only

#### S3.7 Esophago-gastric cancers

| Table 7 Population-based estimates of the frequencies of presenting symptoms among esophago-gastric cancer patients [22–25] | | | | | |
| --- | --- | --- | --- | --- | --- |
| **Study** | **Setting/ source of data** | **Study period** | **Sample size** | **Study population age/range** | **Symptoms** |
| Stephens et al., 2005 ^1^ | Secondary care,  Self-reported and verified with medical records | 1995–03 | 300 | 17–93 years | Weight loss 44.0%  Vomiting 35.7%  Anemia 28.7%  Dysphagia 27.7%  GI bleed 18.3% |
| Hippisley-Cox & Coupland, 2011b | Primary care,  QResearch data (Read coded) | 2000–10 | 2527 | 30–84 years | Dysphagia 32.3%  Haematemesis 7.5%  Abdominal pain 23.0%  Appetite loss 2.6%  Weight loss 8.0% |
| Collins & Altman, 2012b ^2^ | Primary care,  THIN data (Read coded) | 2000–08 | 1343 | 30–84 years | Dysphagia 45.9%  Haematemesis 6.2%  Abdominal pain 24.7%  Appetite loss 2.1%  Weight loss 12.3% |
| Stapley et al., 2013 | Primary care,  CPRD data (Read coded) | 2000–09 | 7471 | 40+ years | Dysphagia 32.4%  Dyspepsia 17.3%  Nausea or vomiting 13.1%  Abdominal pain 12.1% ^3^  Epigastric pain 8.3%  Reflux 11.3%  Chest pain 9.7%  Weight loss 8.2%  Constipation 8.1% |

GI bleed = gastro-intestinal bleed

^1^ only described frequency of alarm symptoms before diagnosis of gastric cancer, and not non-alarm symptoms

^2^ all symptom frequencies calculated manually based on published data; frequencies based on symptoms reported separately for men and women diagnosed with esophago-gastric cancers

^3^ specified as all unspecified abdominal pain excluding epigastric pain

#### S3.8 Ovarian cancer

| Table 8 Population-based estimates of the frequencies of presenting symptoms among ovarian cancer patients [26–29] | | | | | |
| --- | --- | --- | --- | --- | --- |
| **Study** | **Setting/ source of data** | **Study period** | **Sample size** | **Study population age/range** | **Symptoms** |
| Hamilton et al., 2009b | Primary care,  Data from 39 general practices in Exeter/ Devon area | 2000–07 | 212 | 40+ years | Abdominal distension 36% Abdominal bloating 17% Abdominal pain 53% Post-menopausal bleeding 13% Loss of appetite 21% Constipation 20% Diarrhea 27% Rectal bleeding 8.5% Urinary frequency 14% |
| Hippisley-Cox & Coupland, 2012b | Primary care,  QResearch data (coded data only) | 2000–10 | 538 | 30–84 years | Abdominal pain 49.4% Post-menopausal bleeding 9.1% Abdominal distension 7.8% Weight loss 4.1%  Loss of appetite 2% Rectal bleeding 2% |
| Collins & Altman, 2012c | Primary care,  THIN data (Read coded) | 2000–08 | 735 | 30–84 years | Abdominal pain 50.5% Post-menopausal bleeding 9.0% Abdominal distension 11.0% Weight loss 4.8%  Loss of appetite 1.2% Rectal bleeding 3.3% |
| Lim et al., 2015 ^3^ | Subsample of women enrolled in UKOPS ^1^  self-reported symptoms before diagnosis & data from primary care records | 2006–08 | 182 ^2^ | 45+ years | Pelvic or abdominal pain/discomfort 34% 25.1% Increased abdominal size 37.9% 10.8%  Bloating 41.4% 7.4%  Lump in abdomen 8.9% 5.4%  Indigestion 14.3% 7.9%  Constipation 13.8% 6.9%  Diarrhea 7.4% 3.0%  Change in bowel habit 1.0% 6.4%  Nausea or vomiting 8.9% 5.9%  Irregular vaginal bleeding 6.9% 5.9%  Urinary frequency or urgency 16.7% 10.8%  Loss of appetite 13.8% 2.5%  Weight loss 13.8% 3.9%  Fatigue 24.1% 6.9%  Back pain 9.4% 8.9% |

^1^ UK Ovarian Cancer Population Study, a multi-center biobank case-control study

^2^ number of patients who had invasive epithelial (type I and II) (n=158) and borderline ovarian cancer (n=24)

^3^ all symptom frequencies calculated manually based on published data; frequencies based on patient questionnaire and GP notes respectively

#### S3.9 Pancreatic cancer

| Table 9 Population-based estimates of the frequencies of presenting symptoms among pancreatic cancer patients [30,3,31–34] | | | | | |
| --- | --- | --- | --- | --- | --- |
| **Study** | **Setting/ source of data** | **Study period** | **Sample size** | **Study population age/range** | **Symptoms** |
| Stapley et al., 2012 | Primary care,  CPRD data (Read coded) | 2000–09 | 3635 | 40+ years | Abdominal pain 42.4%  Jaundice 30.5%  New onset diabetes 22.1%  Nausea/vomiting 16.2%  Back pain 12.4%  Constipation 11.8%  Diarrhea 10.6%  Weight loss 9.7%  Malaise 5.1% |
| Hippisley-Cox & Coupland, 2012c | Primary care,  QResearch data (Read coded) | 2000–10 | 781 | 30–84 years | Abdominal pain 39.8% Weight loss 7.8%  Appetite loss 3.5% Dysphagia 1.4% Abdominal distension 1.2% |
| Collins & Altman, 2013a ^1^ | Primary care,  THIN data (Read coded) | 2000–08 | 287 | 30–84 years | Abdominal pain 57.3% Appetite loss 3.9% Weight loss 13.3% |
| Keane et al., 2014 | Primary care,  THIN data (Read coded) | 2000–10 | 296 | 18+ years | Abdominal pain 44% Back pain 30% Non-cardiac chest pain 13% Shoulder pain 7% Dyspepsia/reflux 26% Nausea and vomiting 20% Abdominal mass 4% Bloating 3% Upper GI bleeding 3% Dysphagia 2% Hepatomegaly 1% Jaundice 35% Pruritus 8% Change in bowel habit 35% Pancreatitis 4% Steatorrhea 1% Weight loss 10% Lethargy 8% Anorexia 5% DVT/PE 4% Insomnia 2% Fracture 1% Change in taste/smell 0.7% |
| Walter et al., 2016b | Primary & secondary care data; self-reported symptoms before diagnosis | 2010–12 | 391 | 40+ years | Indigestion 27% Decreased appetite 28% Fatigue 20% Feeling different 21% Change in bowel habit 27% Weight loss 16% Back pain 15% Jaundice 12% Change in urine/stool color 11% |
| Price et al., 2016 ^2^ | Primary care, CPRD data (Read coded & uncoded data) | 2000–09 | 3647 | 40+ years | Jaundice 42.9% Abdominal pain 49.1% |

DVT/PE = deep vein thrombosis/pulmonary embolism

^1^ all symptom frequencies calculated manually based on published data; frequencies based on symptoms reported separately for men and women diagnosed with pancreatic cancer

^2^ majority of patients derived from same study population as Stapley et al., 2012 but represents a slightly different analysis encompassing purposefully selected symptoms.

#### S3.10 Prostate cancer

| Table 10 Population-based estimates of the frequencies of presenting symptoms among prostate cancer patients [8,35] | | | | | |
| --- | --- | --- | --- | --- | --- |
| **Study** | **Setting/ source of data** | **Study period** | **Sample size** | **Study population age/range** | **Symptoms** |
| Hamilton et al., 2006 | Primary care, data from 21 general practices in Exeter | 1998–02 | 217 | 40+ years | Retention 15%  Hesitancy 17%  Impotence 31%  Frequency 47%  Nocturia 29%  Hematuria 15% |
| Redaniel et al., 2015 | Primary care, CPRD data (Read coded) | 1998–09 | 1763 | 15+ years | Enlarged prostate 20.2%  Hematuria 33.6%  Hesitancy 4.8%  Nocturia 37.2%  Poor stream 2.1%  Terminal dribbling 2.0% |

#### S3.11 Renal cancer

| Table 11 Population-based estimates of the frequencies of presenting symptoms among renal cancer patients [36–38] | | | | | |
| --- | --- | --- | --- | --- | --- |
| **Study** | **Setting/ source of data** | **Study period** | **Sample size** | **Study population age/range** | **Symptoms** |
| Shephard et al., 2013 | Primary care, CPRD data (Read coded) | 2000–09 | 3149 | 40+ years | Visible hematuria 18% Back pain 11% Abdominal pain 11% Fatigue 7% Constipation 6% Nausea 5% Lower urinary tract infection 11% |
| Hippisley-Cox & Coupland, 2012d ^1^ | Primary care, QResearch data (Read coded) | 2000–10 | 1622 | 30–84 years | Hematuria 74.0% Abdominal pain 11%  Appetite loss 0.4% Weight loss 2.3% Anemia 4.2% |
| Collins & Altman, 2013b ^1^ | Primary care, THIN data (Read coded) | 2000–08 | 2283 | 30–84 years | Hematuria 72.1% Abdominal pain 11%  Appetite loss 0.7% (women only) Weight loss 3.5% (women only) Anemia 4.5% |

^1^ includes bladder cancer cases

## Cancers with a broad symptom signature, low predictive value

#### S3.12 Brain cancer

| Table 12 Population-based estimates of the frequencies of presenting symptoms among brain cancer patients [39] | | | | | |
| --- | --- | --- | --- | --- | --- |
| **Study** | **Setting/ source of data** | **Study period** | **Sample size** | **Study population age/range** | **Symptoms** |
| Hamilton et al., 2007 | Primary care, CPRD data (Read coded) | 1988–06 | 3505 | 18+ years | Headache 10.2% Motor loss 8.8% New onset seizure 4.4% Confusion 3.1% Weakness 2.7% Memory loss 1.1% Visual disorder 1.0% |

#### S3.13 Leukemia

| Table 13 Population-based estimates of the frequencies of presenting symptoms among leukemia patients [40] | | | | | |
| --- | --- | --- | --- | --- | --- |
| **Study** | **Setting/ source of data** | **Study period** | **Sample size** | **Study population age/range** | **Symptoms** |
| Shephard et al., 2016 ^1^ | Primary care, CPRD data (Read coded) | 2000–09 | 937 ^1^ | 40+ years | Infection 25% ^3^  Breathlessness 15%  Fatigue 12%  Chest pain 10%  Abdominal pain 10%  Diarrhea 7%  Vomiting and nausea 6%  Bruising 4% ^4^  Fever 3%  Nosebleeds and bleeding gums 3%  Flu 2%  Weight loss 2% |
| Shephard et al., 2016 ^2^ | Primary care, CPRD data (Read coded) | 2000–09 | 2877 ^2^ | 40+ years | Infection 21% ^3^  Cough 14%  Hypertension 14%  Breathlessness 7% |

^1^ patients with acute leukemia

^2^ patients with chronic leukemia

^3^ infection consists of urinary tract infection, upper respiratory tract infection, skin infection, and chest infection symptoms

^4^ bruising consists of bruising, hematoma, and contusion symptoms

#### S3.14 Lymphoma

| Table 14 Population-based estimates of the frequencies of presenting symptoms among lymphoma patients [41,42] | | | | | |
| --- | --- | --- | --- | --- | --- |
| **Study** | **Setting/ source of data** | **Study period** | **Sample size** | **Study population age/range** | **Symptoms** |
| Shephard et al., 2015a | Primary care, CPRD data (Read coded) | 2000–09 | 283 ^1^ | 40+ years | Lymphadenopathy 18%  Head and neck mass 11% ^3^  Lump 7% |
| Shephard et al., 2015b ^4^ | Primary care, CPRD data (Read coded) | 2000–09 | 4362 ^2^ | 40+ years | Infection 21%  Lymphadenopathy 14% Abdominal pain 14% Mass 11% Shortness of breath 9% Head and neck mass 8% Fatigue 7% Constipation 6% Vomiting and nausea 6%  Indigestion 5%  Weight loss 4% Back pain (re-occurrence) 4%  Malaise 4% |

^1^ Hodgkin’s Lymphoma

^2^ non-Hodgkin’s Lymphoma

^3^ includes cervical lymphadenopathy (enlarged neck lymph nodes)

^4^ infection consists of urinary tract infection, upper respiratory tract infection, skin infection, and chest infection symptoms

#### S3.15 Myeloma

| Table 15 Population-based estimates of the frequencies of presenting symptoms among myeloma patients [43] | | | | | |
| --- | --- | --- | --- | --- | --- |
| **Study** | **Setting/ source of data** | **Study period** | **Sample size** | **Study population age/range** | **Symptoms** |
| Shephard et al., 2015c | Primary care, CPRD data (Read coded) | 2000–09 | 2703 | 40+ years | Back pain 28% Chest pain 15% Chest infection 12% Breathlessness 10% Nausea 6% Fracture 6% Joint pain 4% Combined bone pain 4% Weight loss 4% Rib pain 3% Nose bleeds 3% |

# References

[1] Whiting PF. QUADAS-2: A Revised Tool for the Quality Assessment of Diagnostic Accuracy Studies. Ann Intern Med 2011;155:529. doi:10.7326/0003-4819-155-8-201110180-00009.

[2] Benchimol EI, Smeeth L, Guttmann A, Harron K, Moher D, Petersen I, et al. The REporting of studies Conducted using Observational Routinely-collected health Data (RECORD) Statement. PLoS Med 2015;12:1–22. doi:10.1371/journal.pmed.1001885.

[3] Price SJ, Stapley SA, Shephard E, Barraclough K, Hamilton WT. Is omission of free text records a possible source of data loss and bias in Clinical Practice Research Datalink studies? A case–control study. BMJ Open 2016;6:e011664. doi:10.1136/bmjopen-2016-011664.

[4] Shephard EA, Stapley S, Neal RD, Rose P, Walter FM, Hamilton WT. Clinical features of bladder cancer in primary care. Br J Gen Pract 2012;62:e598-604. doi:10.3399/bjgp12X654560.

[5] Price SJ, Shephard EA, Stapley SA, Barraclough K, Hamilton WT. Non-visible versus visible haematuria and bladder cancer risk: a study of electronic records in primary care. Br J Gen Pract 2014;64:e584-9. doi:10.3399/bjgp14X681409.

[6] Koo MM, Wagner C von, Abel G, McPhail S, Rubin G, Lyratzopoulos G. Typical and atypical symptoms in women with breast cancer: Evidence of variation in diagnostic intervals from a national audit of cancer diagnosis. Cancer Epidemiol 2017;48:140–6. doi:10.1016/j.canep.2017.04.010.

[7] Walker S, Hyde C, Hamilton W. Risk of breast cancer in symptomatic women in primary care: a case-control study using electronic records. Br J Gen Pract 2014;64:e788–93. doi:10.3399/bjgp14X682873.

[8] Redaniel MT, Martin RM, Ridd MJ, Wade J, Jeffreys M. Diagnostic Intervals and Its Association with Breast, Prostate, Lung and Colorectal Cancer Survival in England: Historical Cohort Study Using the Clinical Practice Research Datalink. PLoS One 2015;10:e0126608. doi:10.1371/journal.pone.0126608.

[9] Walker S, Hamilton W. Risk of cervical cancer in symptomatic women aged ≥40 in primary care: A case-control study using electronic records. Eur J Cancer Care (Engl) 2017;63:e12706. doi:10.1111/ecc.12706.

[10] Hamilton W, Round A, Sharp D, Peters TJ. Clinical features of colorectal cancer before diagnosis: a population-based case-control study. Br J Cancer 2005;93:399–405. doi:10.1038/sj.bjc.6602714.

[11] Stapley S, Peters TJ, Sharp D, Hamilton W. The mortality of colorectal cancer in relation to the initial symptom at presentation to primary care and to the duration of symptoms: a cohort study using medical records. Br J Cancer 2006;95:1321–5. doi:10.1038/sj.bjc.6603439.

[12] Hamilton W, Lancashire R, Sharp D, Peters TJ, Cheng K, Marshall T. The risk of colorectal cancer with symptoms at different ages and between the sexes: a case-control study. BMC Med 2009;7:17. doi:10.1186/1741-7015-7-17.

[13] Hippisley-Cox J, Coupland C. Identifying patients with suspected colorectal cancer in primary care: derivation and validation of an algorithm. Br J Gen Pract 2012;62:e29-37. doi:10.3399/bjgp12X616355.

[14] Collins GS, Altman DG. Identifying patients with undetected colorectal cancer: an independent validation of QCancer (Colorectal). Br J Cancer 2012;107:260–5. doi:10.1038/bjc.2012.266.

[15] Walter FM, Emery JD, Mendonca S, Hall N, Morris HC, Mills K, et al. Symptoms and patient factors associated with longer time to diagnosis for colorectal cancer: results from a prospective cohort study. Br J Cancer 2016;115:533–41. doi:10.1038/bjc.2016.221.

[16] Renzi C, Lyratzopoulos G, Card T, Chu TPC, Macleod U, Rachet B. Do colorectal cancer patients diagnosed as an emergency differ from non-emergency patients in their consultation patterns and symptoms? A longitudinal data-linkage study in England. Br J Cancer 2016:1–10. doi:10.1038/bjc.2016.250.

[17] Walker S, Hyde C, Hamilton W. Risk of uterine cancer in symptomatic women in primary care: case-control study using electronic records. Br J Gen Pract 2013;63:e643-8. doi:10.3399/bjgp13X671632.

[18] Walter FM, Rubin G, Bankhead C, Morris HC, Hall N, Mills K, et al. Symptoms and other factors associated with time to diagnosis and stage of lung cancer: a prospective cohort study. Br J Cancer 2015;112:S6-13. doi:10.1038/bjc.2015.30.

[19] Hamilton W, Peters TJ, Round A, Sharp D. What are the clinical features of lung cancer before the diagnosis is made? A population based case-control study. Thorax 2005;60:1059–65. doi:10.1136/thx.2005.045880.

[20] Ades AE, Biswas M, Welton NJ, Hamilton W. Symptom lead time distribution in lung cancer: natural history and prospects for early diagnosis. Int J Epidemiol 2014;43:1865–73. doi:10.1093/ije/dyu174.

[21] Hippisley-Cox J, Coupland C. Identifying patients with suspected lung cancer in primary care : derivation and validation of an algorithm. Br J Gen Pract 2011;61:715–23. doi:10.3399/bjgp11X606627.e715.

[22] Stephens MR, Lewis WG, White S, Blackshaw GRJC, Edwards P, Barry JD, et al. Prognostic significance of alarm symptoms in patients with gastric cancer. Br J Surg 2005;92:840–6. doi:10.1002/bjs.4984.

[23] Hippisley-Cox J, Coupland C. Identifying patients with suspected gastro-oesophageal cancer in primary care: derivation and validation of an algorithm. Br J Gen Pract 2011;61:e707-14. doi:10.3399/bjgp11X606609.

[24] Collins GS, Altman DG. Identifying patients with undetected gastro-oesophageal cancer in primary care: External validation of QCancer® (Gastro-Oesophageal). Eur J Cancer 2013;49:1040–8. doi:10.1016/j.ejca.2012.10.023.

[25] Stapley S, Peters TJ, Neal RD, Rose PW, Walter FM, Hamilton W. The risk of oesophago-gastric cancer in symptomatic patients in primary care: a large case-control study using electronic records. Br J Cancer 2013;108:25–31. doi:10.1038/bjc.2012.551.

[26] Hamilton W, Peters TJ, Bankhead C, Sharp D. Risk of ovarian cancer in women with symptoms in primary care: population based case-control study. BMJ 2009;339:b2998. doi:10.1136/bmj.b2998.

[27] Hippisley-Cox J, Coupland C. Identifying women with suspected ovarian cancer in primary care: derivation and validation of algorithm. BMJ 2011;344:d8009–d8009. doi:10.1136/bmj.d8009.

[28] Collins GS, Altman DG. Identifying women with undetected ovarian cancer: independent and external validation of QCancer ® (Ovarian) prediction model. Eur J Cancer Care (Engl) 2013;22:423–9. doi:10.1111/ecc.12015.

[29] Lim A, Mesher D, Gentry-Maharaj A, Balogun N, Widschwendter M, Jacobs I, et al. Time to diagnosis of Type I or II invasive epithelial ovarian cancers: a multicentre observational study using patient questionnaire and primary care records. BJOG 2015:1–9. doi:10.1111/1471-0528.13447.

[30] Walter FM, Mills K, Mendonça SC, Abel GA, Basu B, Carroll N, et al. Symptoms and patient factors associated with diagnostic intervals for pancreatic cancer (SYMPTOM pancreatic study): a prospective cohort study. Lancet Gastroenterol Hepatol 2016;1253:1–9. doi:10.1016/S2468-1253(16)30079-6.

[31] Stapley S, Peters TJ, Neal RD, Rose PW, Walter FM, Hamilton W. The risk of pancreatic cancer in symptomatic patients in primary care: a large case-control study using electronic records. Br J Cancer 2012;106:1940–4. doi:10.1038/bjc.2012.190.

[32] Hippisley-Cox J, Coupland C. Identifying patients with suspected pancreatic cancer in primary care: derivation and validation of an algorithm. Br J Gen Pract 2012;62:38–45. doi:10.3399/bjgp12X616355.

[33] Collins GS, Altman DG. Identifying patients with undetected pancreatic cancer in primary care: an independent and external validation of QCancer® (Pancreas). Br J Gen Pract 2013;63:636–42. doi:10.3399/bjgp13X671623.

[34] Keane MG, Horsfall L, Rait G, Pereira SP. A case-control study comparing the incidence of early symptoms in pancreatic and biliary tract cancer. BMJ Open 2014;4:e005720–e005720. doi:10.1136/bmjopen-2014-005720.

[35] Hamilton W, Sharp DJ, Peters TJ, Round AP. Clinical features of prostate cancer before diagnosis: a population-based, case-control study. Br J Gen Pract 2006;56:756–62.

[36] Shephard E, Neal R, Rose P, Walter F, Hamilton WT. Clinical features of kidney cancer in primary care: a case-control study using primary care records. Br J Gen Pract 2013;63:e250-5. doi:10.3399/bjgp13X665215.

[37] Hippisley-Cox J, Coupland C. Identifying patients with suspected renal tract cancer in primary care : derivation and validation of an algorithm 2012:251–60. doi:10.3399/bjgp12X636074.e251.

[38] Collins GS, Altman DG. Identifying patients with undetected renal tract cancer in primary care: an independent and external validation of QCancer® (Renal) prediction model. Cancer Epidemiol 2013;37:115–20. doi:10.1016/j.canep.2012.11.005.

[39] Hamilton W, Kernick D. Clinical features of primary brain tumours: a case-control study using electronic primary care records. Br J Gen Pract 2007;57:695–9.

[40] Shephard EA, Neal RD, Rose PW, Walter FM, Hamilton W. Symptoms of adult chronic and acute leukaemia before diagnosis: large primary care case-control studies using electronic records. Br J Gen Pract 2016;66:e182–8. doi:10.3399/bjgp16X683989.

[41] Shephard EA, Neal RD, Rose PW, Walter FM, Hamilton WT. Quantifying the risk of Hodgkin lymphoma in symptomatic primary care patients aged ≥40 years: a case-control study using electronic records. Br J Gen Pract 2015;65:e289-94. doi:10.3399/bjgp15X684793.

[42] Shephard EA, Neal RD, Rose PW, Walter FM, Hamilton WT. Quantifying the risk of non-Hodgkin lymphoma in symptomatic primary care patients aged ≥40 years: a large case-control study using electronic records. Br J Gen Pract 2015;65:e281-8. doi:10.3399/bjgp15X684793.

[43] Shephard EA, Neal RD, Rose P, Walter FM, Litt EJ, Hamilton WT. Quantifying the risk of multiple myeloma from symptoms reported in primary care patients: a large case-control study using electronic records. Br J Gen Pract 2015;65:e106–13. doi:10.3399/bjgp15X683545.
